# {2,7-Dieth­oxy-8-[(naphthalen-1-yl)carbon­yl]naph­thalen-1-yl}(naphthalen-1-yl)methanone

**DOI:** 10.1107/S1600536813005710

**Published:** 2013-03-06

**Authors:** Takehiro Tsumuki, Ryo Takeuchi, Hiroyuki Kawano, Noriyuki Yonezawa, Akiko Okamoto

**Affiliations:** a Department of Organic and Polymer Materials Chemistry, Tokyo University of Agriculture & Technology, Koganei, Tokyo 184-8588, Japan; b Division of Liberal Arts, Kogakuin University, Hachioji, Tokyo 192-0015, Japan

## Abstract

In the title compound, C_36_H_28_O_4_, the 1-naphthoyl groups at the 1- and 8-positions of the central 2,7-dieth­oxy­naphthalene ring system are aligned almost anti­parallel and make a dihedral angle of 76.59 (4)°. The dihedral angles between the central 2,7-dieth­oxy­naphthalene ring system and the terminal naphthalene ring systems are 86.48 (4) and 83.97 (4)°. In the crystal, C—H⋯π inter­actions between the central naphthalene ring systems and the naphthoyl groups are observed along the *a* axis, with the mol­ecules forming a columnar structure. The columns are linked into chains parallel to the *b* axis by C—H⋯O inter­actions.

## Related literature

For electrophilic aroylation of naphthalene derivatives, see: Okamoto & Yonezawa (2009 ▶); Okamoto *et al.* (2011 ▶). For the structures of closely related compounds, see: Nakaema *et al.* (2008 ▶); Tsumuki *et al.* (2011 ▶); Sakamoto *et al.* (2012 ▶); Isogai *et al.* (2013 ▶); Tsumuki *et al.* (2013 ▶); Yoshiwaka *et al.* (2013 ▶).
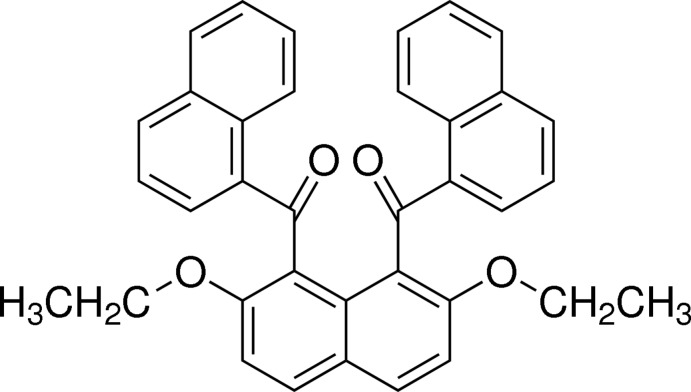


## Experimental

### 

#### Crystal data

C_36_H_28_O_4_
*M* *_r_* = 524.58
Triclinic, 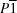

*a* = 8.76532 (16) Å
*b* = 11.4266 (2) Å
*c* = 14.1972 (3) Å
α = 99.080 (1)°
β = 99.036 (1)°
γ = 104.277 (1)°
*V* = 1331.94 (4) Å^3^
*Z* = 2
Cu *K*α radiation
μ = 0.67 mm^−1^
*T* = 193 K
0.60 × 0.40 × 0.20 mm

#### Data collection

Rigaku R-AXIS RAPID diffractometer
Absorption correction: numerical (*NUMABS*; Higashi, 1999 ▶) *T*_min_ = 0.689, *T*_max_ = 0.877
24143 measured reflections
4800 independent reflections
4142 reflections with *I* > 2σ(*I*)
*R*_int_ = 0.043

#### Refinement

*R*[*F* ^2^ > 2σ(*F* ^2^)] = 0.037
*wR*(*F* ^2^) = 0.106
*S* = 1.07
4800 reflections
364 parameters
H-atom parameters constrained
Δρ_max_ = 0.20 e Å^−3^
Δρ_min_ = −0.16 e Å^−3^

### 

Data collection: *PROCESS-AUTO* (Rigaku, 1998 ▶); cell refinement: *PROCESS-AUTO*; data reduction: *PROCESS-AUTO*; program(s) used to solve structure: *Il Milione* (Burla *et al.*, 2007 ▶); program(s) used to refine structure: *SHELXL97* (Sheldrick, 2008 ▶); molecular graphics: *ORTEPIII* (Burnett & Johnson, 1996 ▶); software used to prepare material for publication: *SHELXL97*.

## Supplementary Material

Crystal structure: contains datablock(s) I, global. DOI: 10.1107/S1600536813005710/pk2467sup1.cif

Structure factors: contains datablock(s) I. DOI: 10.1107/S1600536813005710/pk2467Isup2.hkl

Supplementary material file. DOI: 10.1107/S1600536813005710/pk2467Isup3.cml

Additional supplementary materials:  crystallographic information; 3D view; checkCIF report

## Figures and Tables

**Table 1:** Hydrogen-bond geometry (Å, °) *Cg*4 and *Cg*6 are the centroids of the C16–C21 and C27–C32 rings, respectively.

*D*—H⋯*A*	*D*—H	H⋯*A*	*D*⋯*A*	*D*—H⋯*A*
C3—H3⋯*Cg*4^i^	0.95	2.77	3.5662 (15)	142
C7—H7⋯*Cg*6^i^	0.95	2.76	3.5662 (16)	143
C30—H30⋯O2^ii^	0.95	2.53	3.3289 (19)	142
C34—H34*A*⋯O1^iii^	0.98	2.47	3.423 (2)	163
C35—H35*B*⋯O2^iv^	0.99	2.59	3.5476 (17)	163

Symmetry codes: (i) 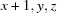; (ii) 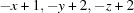; (iii) 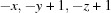; (iv) 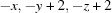.

**Fig. 1.**
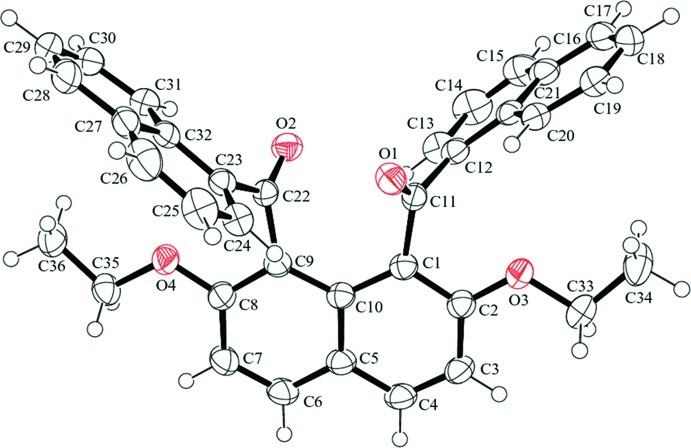
The molecular structure of the title compound, with displacement ellipsoids drawn at the 50% probability level.

**Fig. 2.**
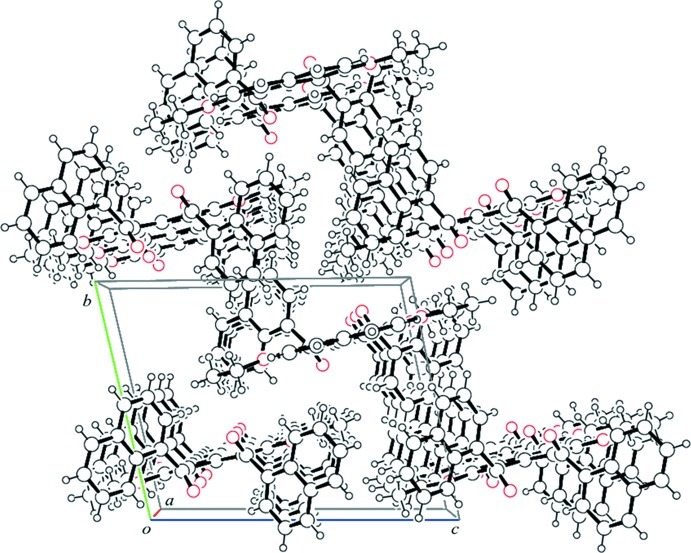
The arrangement of the molecules in the crystal structure, viewed down the a axis.

**Fig. 3.**
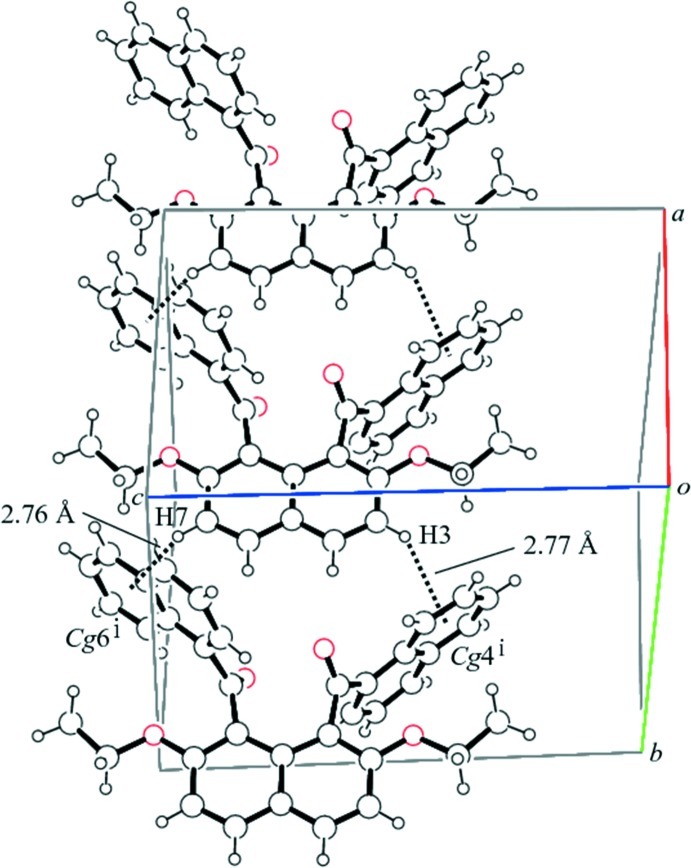
A partial view of the crystal packing of the title compound, showing the intermolecular C—H···π interactions. Cg4 and Cg6 are centroid of the C16–C21 and C27–C32 (see Table 1 for details; symmetry codes: (i) 1 + x, y, z).

**Fig. 4.**
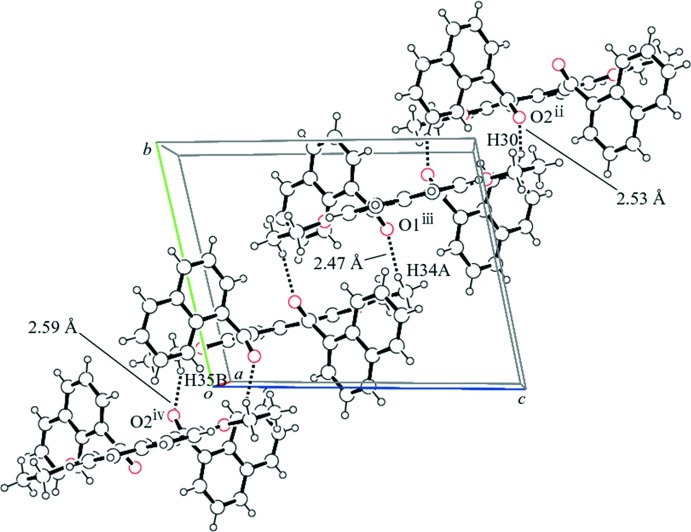
A partial view of the crystal packing of the title compound, showing the intermolecular C—H···O interactions (see Table 1 for details; symmetry codes: (ii) 1 - x, 2 - y, 2 - z; (iii) - x, - 1 - y, - 1 - z (iv); - x, 2 - y, 2 - z).

## supplementary crystallographic information

### Comment

In the course of our study on selective electrophilic aromatic aroylation of the naphthalene ring core, 1,8-diaroylnaphthalene compounds have proved to be formed regioselectively by the aid of a suitable acidic mediator (Okamoto & Yonezawa, 2009, Okamoto *et al.*, 2011). Recently, we have reported the crystal structures of several 1,8-diaroylated naphthalene analogues exemplified by 1,8-dibenzoyl-2,7-dimethoxynaphthalene (Nakaema *et al.*, 2008) and [2,7-dimethoxy-8-(2-naphthoyl)naphthalen-1-yl](naphthalen-2-yl)methanone (Tsumuki *et al.*, 2011). Furthermore, crystal structures of 1,8-diaroylnaphthalene analogues bearing various alkoxy and aryloxy groups at the 2,7-positions such as 1,8-dibenzoylnaphthalene-2,7-diyl dibenzoate (Sakamoto *et al.*, 2012) and [8-(4-phenoxybenzoyl)-2,7-bis(propan-2-yloxy)naphthalen-1-yl](4-phenoxyphenyl)methanone (Yoshiwaka *et al.*, 2013) have been also revealed. Some 1,8-diaroylnaphthalene compounds bearing the ethoxy group, {2,7-diethoxy-8-[(naphthalen-2-yl)-carbonyl]naphthalen-1-yl}(naphthalen-2-yl)methanone (Tsumuki *et al.*, 2013) and (8-benzoyl-2,7-diethoxynaphthalen-1-yl)(phenyl)methanone (Isogai *et al.*, 2013), are stabilized by the molecular packing of C—H···O interactions between the aroyl groups. As a part of our ongoing studies on the molecular structures of these kinds of homologous molecules, the X-ray crystal structure of the title compound, the 2,7-diethoxynaphthalene bearing *α*-naphthoyl groups at the 1,8-positions, is reported on herein.

The molecular structure of the title molecule is illustrated in Fig.1. The two terminal naphthoyl groups are oriented in opposite directions and are twisted away from the central 2,7-diethoxynaphthalene unit. The carbonyl moieties deviate slightly from the attached naphthalene rings. The dihedral angle between the two naphthalene rings of the terminal naphthoyl groups (C12–C21 and C23–C32) is 76.59 (4)°. The dihedral angles between the terminal naphthalene rings and the central naphthalene ring (C1–C10) are 86.48 (4) and 83.97 (4)°. The torsion angles between the carbonyl moieties and the central naphthalene ring are -60.91 (16)° (C10—C1—C11—O1) and -65.50 (17)° (C10—C9—C22—O2), and those between the carbonyl moieties and the terminal naphthalene rings are -47.50 (17)° (O1—C11—C12—C21) and -46.38 (17)° (O2—C22—C23—C32).

In the molecular packing, C—H···π interactions between the central naphthalene rings and the naphthoyl groups are observed along the *a* axis, and form columnar structures (Fig. 2, 3 and Table 1). Each column is linked into chains along the *b* axis by C—H···O interactions (Fig. 4 and Table 1).

### Experimental

To a solution of 1-naphthoyl chloride (630 mg, 3.3 mmol) and TiCl_4_ (1.88 g, 9.9 mmol) in CH_2_Cl_2_ (2.5 ml), 2,7-diethoxynaphthalene (220 mg, 1.0 mmol) was added. The reaction mixture was stirred at r.t. for 3 h, then poured into ice-cold water (20 ml). The aqueous layer was extracted with CHCl_3_ (20 ml × 3). The combined organic extracts were washed with 2 *M* aqueous NaOH (25 ml × 3) followed by washing with brine (25 ml × 3). The organic layer was dried over anhydrous MgSO_4_. The solvent was removed under reduced pressure to give a cake (yield 95%). The crude product was purified by recrystallization from chloroform (isolated yield 60%). Colorless platelet single crystals suitable for X-ray diffraction were obtained by repeated crystallization from chloroform.

^1^H NMR δ (500 MHz, CDCl_3_): 0.57 (6*H*, broad), 3.78 (4*H*, broad), 7.13 (2*H*, d, *J* = 9.0 Hz), 7.27–7.33 (6*H*, m), 7.71–7.83 (6*H*, m), 7.91 (2*H*, d, *J* = 9.0 Hz), 8.15 (2*H*, broad) p.p.m.; ^13^C NMR δ (125 MHz, CDCl_3_): 14.12, 64.99, 112.73, 124.29, 124.70,125.53, 125.65, 126.45, 127.26, 127.88, 130.31, 130.52, 130.85, 132.33, 132.39,133.61, 137.15, 156.87, 199.49 p.p.m.; IR (KBr): 1658, 1607, 1512, 1471, 1275 cm^-1^; HRMS (*m/z*): [*M*+H]^+^ calcd. for C_36_H_29_O_4_, 525.2066, found, 525.2032.

### Refinement

All the H atoms were located in a difference Fourier map and were subsequently refined as riding atoms: C—H = 0.95 (aromatic), 0.98 (methyl) and 0.99 (methylene) Å, with *U*iso(H) = 1.2 *U*eq(C).

### Crystal data

**Table:**

C_36_H_28_O_4_	*Z* = 2
*M**_r_* = 524.58	*F*(000) = 552
Triclinic, *P*1	*D*_x_ = 1.308 Mg m^−^^3^
Hall symbol: -P 1	Melting point = 506.6–508.4 K
*a* = 8.76532 (16) Å	Cu *K*α radiation, λ = 1.54187 Å
*b* = 11.4266 (2) Å	Cell parameters from 20940 reflections
*c* = 14.1972 (3) Å	θ = 3.2–68.2°
α = 99.080 (1)°	µ = 0.67 mm^−^^1^
β = 99.036 (1)°	*T* = 193 K
γ = 104.277 (1)°	Platelet, colorless
*V* = 1331.94 (4) Å^3^	0.60 × 0.40 × 0.20 mm

### Data collection

**Table:**

Rigaku R-AXIS RAPID diffractometer	4800 independent reflections
Radiation source: rotating anode	4142 reflections with *I* > 2σ(*I*)
Graphite monochromator	*R*_int_ = 0.043
Detector resolution: 10.000 pixels mm^-1^	θ_max_ = 68.2°, θ_min_ = 3.2°
ω scans	*h* = −10→10
Absorption correction: numerical (*NUMABS*; Higashi, 1999)	*k* = −13→13
*T*_min_ = 0.689, *T*_max_ = 0.877	*l* = −17→17
24143 measured reflections	

### Refinement

**Table:**

Refinement on *F*^2^	Secondary atom site location: difference Fourier map
Least-squares matrix: full	Hydrogen site location: inferred from neighbouring sites
*R*[*F*^2^ > 2σ(*F*^2^)] = 0.037	H-atom parameters constrained
*wR*(*F*^2^) = 0.106	*w* = 1/[σ^2^(*F*_o_^2^) + (0.058*P*)^2^ + 0.2087*P*] where *P* = (*F*_o_^2^ + 2*F*_c_^2^)/3
*S* = 1.07	(Δ/σ)_max_ = 0.001
4800 reflections	Δρ_max_ = 0.20 e Å^−^^3^
364 parameters	Δρ_min_ = −0.16 e Å^−^^3^
0 restraints	Extinction correction: *SHELXL97* (Sheldrick, 2008), Fc^*^=kFc[1+0.001xFc^2^λ^3^/sin(2θ)]^-1/4^
Primary atom site location: structure-invariant direct methods	Extinction coefficient: 0.0072 (5)

### Special details

**Table:**

Geometry. All e.s.d.'s (except the e.s.d. in the dihedral angle between two l.s. planes) are estimated using the full covariance matrix. The cell e.s.d.'s are taken into account individually in the estimation of e.s.d.'s in distances, angles and torsion angles; correlations between e.s.d.'s in cell parameters are only used when they are defined by crystal symmetry. An approximate (isotropic) treatment of cell e.s.d.'s is used for estimating e.s.d.'s involving l.s. planes.
Refinement. Refinement of *F*^2^ against ALL reflections. The weighted *R*-factor *wR* and goodness of fit *S* are based on *F*^2^, conventional *R*-factors *R* are based on *F*, with *F* set to zero for negative *F*^2^. The threshold expression of *F*^2^ > σ(*F*^2^) is used only for calculating *R*-factors(gt) *etc*. and is not relevant to the choice of reflections for refinement. *R*-factors based on *F*^2^ are statistically about twice as large as those based on *F*, and *R*- factors based on ALL data will be even larger.

### Fractional atomic coordinates and isotropic or equivalent isotropic displacement parameters (Å^2^)

**Table:**

	*x*	*y*	*z*	*U*_iso_*/*U*_eq_	
O1	0.08739 (10)	0.64809 (8)	0.66867 (6)	0.0379 (2)	
O2	0.19599 (10)	0.86929 (8)	0.83185 (6)	0.0388 (2)	
O3	−0.20151 (10)	0.67148 (9)	0.48478 (6)	0.0419 (2)	
O4	−0.01872 (10)	0.83966 (9)	1.00812 (6)	0.0429 (2)	
C1	−0.13726 (14)	0.73032 (10)	0.65387 (9)	0.0312 (3)	
C2	−0.25357 (15)	0.70050 (11)	0.56859 (9)	0.0345 (3)	
C3	−0.41501 (15)	0.69700 (12)	0.56983 (10)	0.0399 (3)	
H3	−0.4924	0.6770	0.5106	0.048*	
C4	−0.45851 (15)	0.72264 (12)	0.65675 (10)	0.0407 (3)	
H4	−0.5671	0.7211	0.6576	0.049*	
C5	−0.34659 (14)	0.75145 (11)	0.74590 (9)	0.0360 (3)	
C6	−0.39763 (15)	0.77691 (13)	0.83424 (10)	0.0429 (3)	
H6	−0.5073	0.7742	0.8325	0.052*	
C7	−0.29423 (16)	0.80531 (13)	0.92212 (10)	0.0430 (3)	
H7	−0.3305	0.8231	0.9810	0.052*	
C8	−0.13216 (15)	0.80769 (12)	0.92383 (9)	0.0364 (3)	
C9	−0.07595 (14)	0.78095 (11)	0.83942 (9)	0.0313 (3)	
C10	−0.18165 (14)	0.75431 (10)	0.74623 (9)	0.0313 (3)	
C11	0.02752 (14)	0.72402 (10)	0.63903 (8)	0.0306 (3)	
C12	0.11099 (14)	0.81261 (11)	0.58353 (9)	0.0327 (3)	
C13	0.11822 (17)	0.93448 (12)	0.61063 (10)	0.0440 (3)	
H13	0.0676	0.9596	0.6617	0.053*	
C14	0.1996 (2)	1.02299 (13)	0.56390 (13)	0.0560 (4)	
H14	0.2063	1.1076	0.5848	0.067*	
C15	0.26881 (18)	0.98776 (14)	0.48875 (12)	0.0530 (4)	
H15	0.3246	1.0484	0.4581	0.064*	
C16	0.25873 (15)	0.86244 (12)	0.45584 (9)	0.0402 (3)	
C17	0.31914 (16)	0.82289 (15)	0.37281 (10)	0.0489 (4)	
H17	0.3743	0.8826	0.3412	0.059*	
C18	0.29954 (16)	0.70146 (15)	0.33786 (10)	0.0481 (4)	
H18	0.3374	0.6766	0.2809	0.058*	
C19	0.22326 (15)	0.61257 (13)	0.38595 (9)	0.0427 (3)	
H19	0.2106	0.5277	0.3615	0.051*	
C20	0.16714 (14)	0.64674 (11)	0.46737 (9)	0.0351 (3)	
H20	0.1180	0.5853	0.4997	0.042*	
C21	0.18097 (13)	0.77222 (11)	0.50452 (8)	0.0325 (3)	
C22	0.10078 (14)	0.78898 (10)	0.85622 (8)	0.0307 (3)	
C23	0.15239 (14)	0.69517 (11)	0.90702 (8)	0.0321 (3)	
C24	0.06855 (16)	0.57328 (11)	0.87330 (9)	0.0385 (3)	
H24	−0.0238	0.5518	0.8222	0.046*	
C25	0.11646 (19)	0.47971 (13)	0.91269 (11)	0.0484 (3)	
H25	0.0583	0.3957	0.8873	0.058*	
C26	0.2463 (2)	0.50928 (14)	0.98733 (11)	0.0515 (4)	
H26	0.2797	0.4455	1.0128	0.062*	
C27	0.33248 (16)	0.63426 (14)	1.02763 (9)	0.0426 (3)	
C28	0.46076 (18)	0.66683 (18)	1.10997 (11)	0.0558 (4)	
H28	0.4948	0.6036	1.1360	0.067*	
C29	0.53555 (17)	0.78660 (18)	1.15211 (10)	0.0583 (4)	
H29	0.6198	0.8066	1.2080	0.070*	
C30	0.48893 (16)	0.88165 (16)	1.11340 (10)	0.0518 (4)	
H30	0.5410	0.9654	1.1437	0.062*	
C31	0.36913 (15)	0.85385 (13)	1.03242 (9)	0.0408 (3)	
H31	0.3408	0.9188	1.0061	0.049*	
C32	0.28633 (14)	0.72967 (12)	0.98702 (8)	0.0351 (3)	
C33	−0.31667 (16)	0.62668 (13)	0.39438 (9)	0.0426 (3)	
H33A	−0.3755	0.6882	0.3820	0.051*	
H33B	−0.3954	0.5489	0.3965	0.051*	
C34	−0.2252 (2)	0.60500 (16)	0.31584 (10)	0.0560 (4)	
H34A	−0.1664	0.5449	0.3294	0.067*	
H34B	−0.1490	0.6829	0.3138	0.067*	
H34C	−0.3005	0.5730	0.2529	0.067*	
C35	−0.06724 (16)	0.86450 (12)	1.09918 (9)	0.0395 (3)	
H35A	−0.1508	0.7922	1.1063	0.047*	
H35B	−0.1118	0.9364	1.1028	0.047*	
C36	0.07978 (18)	0.89118 (14)	1.17804 (10)	0.0472 (3)	
H36A	0.1255	0.8209	1.1717	0.057*	
H36B	0.0504	0.9048	1.2419	0.057*	
H36C	0.1595	0.9652	1.1721	0.057*	

### Atomic displacement parameters (Å^2^)

**Table:**

	*U*^11^	*U*^22^	*U*^33^	*U*^12^	*U*^13^	*U*^23^
O1	0.0401 (5)	0.0440 (5)	0.0379 (5)	0.0200 (4)	0.0118 (4)	0.0157 (4)
O2	0.0334 (5)	0.0402 (5)	0.0419 (5)	0.0070 (4)	0.0067 (4)	0.0117 (4)
O3	0.0345 (5)	0.0570 (6)	0.0308 (5)	0.0128 (4)	0.0012 (4)	0.0042 (4)
O4	0.0364 (5)	0.0645 (6)	0.0302 (5)	0.0211 (4)	0.0071 (4)	0.0055 (4)
C1	0.0292 (6)	0.0307 (6)	0.0343 (6)	0.0085 (5)	0.0052 (5)	0.0093 (5)
C2	0.0336 (6)	0.0352 (6)	0.0346 (6)	0.0094 (5)	0.0049 (5)	0.0094 (5)
C3	0.0307 (6)	0.0474 (7)	0.0384 (7)	0.0080 (5)	−0.0011 (5)	0.0119 (6)
C4	0.0268 (6)	0.0497 (8)	0.0474 (7)	0.0108 (5)	0.0055 (5)	0.0169 (6)
C5	0.0287 (6)	0.0412 (7)	0.0405 (7)	0.0112 (5)	0.0070 (5)	0.0132 (5)
C6	0.0296 (6)	0.0597 (8)	0.0467 (8)	0.0186 (6)	0.0125 (5)	0.0177 (6)
C7	0.0386 (7)	0.0600 (8)	0.0387 (7)	0.0223 (6)	0.0144 (5)	0.0144 (6)
C8	0.0337 (6)	0.0440 (7)	0.0345 (6)	0.0150 (5)	0.0059 (5)	0.0108 (5)
C9	0.0301 (6)	0.0331 (6)	0.0337 (6)	0.0122 (5)	0.0073 (5)	0.0091 (5)
C10	0.0292 (6)	0.0313 (6)	0.0353 (6)	0.0099 (5)	0.0059 (5)	0.0104 (5)
C11	0.0311 (6)	0.0336 (6)	0.0257 (5)	0.0091 (5)	0.0033 (4)	0.0041 (5)
C12	0.0274 (6)	0.0352 (6)	0.0343 (6)	0.0083 (5)	0.0016 (5)	0.0088 (5)
C13	0.0447 (7)	0.0376 (7)	0.0500 (8)	0.0126 (6)	0.0082 (6)	0.0093 (6)
C14	0.0584 (9)	0.0336 (7)	0.0743 (11)	0.0085 (6)	0.0098 (8)	0.0168 (7)
C15	0.0466 (8)	0.0470 (8)	0.0667 (10)	0.0039 (6)	0.0119 (7)	0.0298 (7)
C16	0.0292 (6)	0.0499 (8)	0.0415 (7)	0.0067 (5)	0.0026 (5)	0.0206 (6)
C17	0.0341 (7)	0.0743 (10)	0.0429 (8)	0.0104 (6)	0.0093 (6)	0.0312 (7)
C18	0.0372 (7)	0.0768 (11)	0.0331 (7)	0.0175 (7)	0.0084 (5)	0.0155 (7)
C19	0.0342 (7)	0.0552 (8)	0.0363 (7)	0.0126 (6)	0.0048 (5)	0.0044 (6)
C20	0.0283 (6)	0.0410 (7)	0.0340 (6)	0.0067 (5)	0.0042 (5)	0.0084 (5)
C21	0.0233 (5)	0.0415 (7)	0.0317 (6)	0.0071 (5)	0.0006 (4)	0.0128 (5)
C22	0.0300 (6)	0.0348 (6)	0.0265 (6)	0.0098 (5)	0.0049 (5)	0.0034 (5)
C23	0.0303 (6)	0.0391 (6)	0.0311 (6)	0.0136 (5)	0.0102 (5)	0.0089 (5)
C24	0.0417 (7)	0.0396 (7)	0.0356 (6)	0.0119 (5)	0.0100 (5)	0.0086 (5)
C25	0.0640 (9)	0.0384 (7)	0.0488 (8)	0.0193 (6)	0.0170 (7)	0.0132 (6)
C26	0.0662 (10)	0.0569 (9)	0.0508 (8)	0.0367 (8)	0.0221 (7)	0.0259 (7)
C27	0.0406 (7)	0.0661 (9)	0.0345 (7)	0.0275 (6)	0.0158 (5)	0.0210 (6)
C28	0.0453 (8)	0.0967 (13)	0.0415 (8)	0.0351 (8)	0.0136 (6)	0.0318 (8)
C29	0.0339 (7)	0.1093 (14)	0.0337 (7)	0.0193 (8)	0.0065 (6)	0.0219 (8)
C30	0.0329 (7)	0.0773 (10)	0.0369 (7)	0.0036 (7)	0.0087 (6)	0.0046 (7)
C31	0.0311 (6)	0.0548 (8)	0.0358 (7)	0.0099 (6)	0.0092 (5)	0.0079 (6)
C32	0.0309 (6)	0.0505 (7)	0.0303 (6)	0.0171 (5)	0.0125 (5)	0.0116 (5)
C33	0.0426 (7)	0.0432 (7)	0.0364 (7)	0.0109 (6)	−0.0042 (5)	0.0059 (5)
C34	0.0647 (10)	0.0738 (10)	0.0340 (7)	0.0399 (8)	−0.0017 (6)	0.0034 (7)
C35	0.0448 (7)	0.0451 (7)	0.0345 (7)	0.0209 (6)	0.0129 (6)	0.0074 (5)
C36	0.0519 (8)	0.0569 (8)	0.0339 (7)	0.0207 (7)	0.0083 (6)	0.0042 (6)

### Geometric parameters (Å, º)

**Table:**

O1—C11	1.2148 (14)	C18—H18	0.9500
O2—C22	1.2131 (14)	C19—C20	1.3644 (18)
O3—C2	1.3617 (15)	C19—H19	0.9500
O3—C33	1.4350 (14)	C20—C21	1.4157 (18)
O4—C8	1.3650 (15)	C20—H20	0.9500
O4—C35	1.4315 (15)	C22—C23	1.5015 (16)
C1—C2	1.3901 (16)	C23—C24	1.3723 (17)
C1—C10	1.4301 (17)	C23—C32	1.4266 (16)
C1—C11	1.5093 (16)	C24—C25	1.4031 (18)
C2—C3	1.4088 (18)	C24—H24	0.9500
C3—C4	1.3576 (19)	C25—C26	1.361 (2)
C3—H3	0.9500	C25—H25	0.9500
C4—C5	1.4113 (17)	C26—C27	1.418 (2)
C4—H4	0.9500	C26—H26	0.9500
C5—C6	1.4081 (18)	C27—C28	1.419 (2)
C5—C10	1.4373 (17)	C27—C32	1.4239 (18)
C6—C7	1.3621 (19)	C28—C29	1.355 (2)
C6—H6	0.9500	C28—H28	0.9500
C7—C8	1.4104 (18)	C29—C30	1.409 (2)
C7—H7	0.9500	C29—H29	0.9500
C8—C9	1.3843 (17)	C30—C31	1.3661 (19)
C9—C10	1.4319 (16)	C30—H30	0.9500
C9—C22	1.5073 (16)	C31—C32	1.4185 (19)
C11—C12	1.4994 (16)	C31—H31	0.9500
C12—C13	1.3678 (18)	C33—C34	1.496 (2)
C12—C21	1.4283 (17)	C33—H33A	0.9900
C13—C14	1.406 (2)	C33—H33B	0.9900
C13—H13	0.9500	C34—H34A	0.9800
C14—C15	1.363 (2)	C34—H34B	0.9800
C14—H14	0.9500	C34—H34C	0.9800
C15—C16	1.411 (2)	C35—C36	1.5008 (19)
C15—H15	0.9500	C35—H35A	0.9900
C16—C17	1.421 (2)	C35—H35B	0.9900
C16—C21	1.4251 (17)	C36—H36A	0.9800
C17—C18	1.356 (2)	C36—H36B	0.9800
C17—H17	0.9500	C36—H36C	0.9800
C18—C19	1.404 (2)		
			
C2—O3—C33	119.13 (10)	C21—C20—H20	119.5
C8—O4—C35	118.97 (10)	C20—C21—C16	118.15 (12)
C2—C1—C10	119.84 (11)	C20—C21—C12	123.61 (10)
C2—C1—C11	114.61 (10)	C16—C21—C12	118.08 (11)
C10—C1—C11	125.36 (10)	O2—C22—C23	122.19 (10)
O3—C2—C1	115.41 (11)	O2—C22—C9	121.24 (10)
O3—C2—C3	122.72 (11)	C23—C22—C9	116.54 (10)
C1—C2—C3	121.84 (12)	C24—C23—C32	120.11 (11)
C4—C3—C2	119.11 (11)	C24—C23—C22	118.12 (11)
C4—C3—H3	120.4	C32—C23—C22	121.76 (11)
C2—C3—H3	120.4	C23—C24—C25	121.34 (13)
C3—C4—C5	121.73 (12)	C23—C24—H24	119.3
C3—C4—H4	119.1	C25—C24—H24	119.3
C5—C4—H4	119.1	C26—C25—C24	119.97 (13)
C6—C5—C4	119.63 (11)	C26—C25—H25	120.0
C6—C5—C10	120.35 (11)	C24—C25—H25	120.0
C4—C5—C10	120.01 (12)	C25—C26—C27	120.78 (12)
C7—C6—C5	121.85 (12)	C25—C26—H26	119.6
C7—C6—H6	119.1	C27—C26—H26	119.6
C5—C6—H6	119.1	C26—C27—C28	121.37 (13)
C6—C7—C8	118.56 (12)	C26—C27—C32	119.59 (12)
C6—C7—H7	120.7	C28—C27—C32	119.00 (14)
C8—C7—H7	120.7	C29—C28—C27	121.08 (14)
O4—C8—C9	115.18 (11)	C29—C28—H28	119.5
O4—C8—C7	122.69 (11)	C27—C28—H28	119.5
C9—C8—C7	122.11 (11)	C28—C29—C30	120.29 (13)
C8—C9—C10	120.16 (11)	C28—C29—H29	119.9
C8—C9—C22	114.25 (10)	C30—C29—H29	119.9
C10—C9—C22	125.53 (10)	C31—C30—C29	120.27 (15)
C1—C10—C9	125.67 (11)	C31—C30—H30	119.9
C1—C10—C5	117.42 (11)	C29—C30—H30	119.9
C9—C10—C5	116.91 (11)	C30—C31—C32	121.15 (14)
O1—C11—C12	121.76 (11)	C30—C31—H31	119.4
O1—C11—C1	121.13 (10)	C32—C31—H31	119.4
C12—C11—C1	117.07 (10)	C31—C32—C27	118.16 (12)
C13—C12—C21	120.45 (11)	C31—C32—C23	123.58 (11)
C13—C12—C11	117.95 (11)	C27—C32—C23	118.12 (12)
C21—C12—C11	121.60 (10)	O3—C33—C34	107.15 (11)
C12—C13—C14	120.83 (14)	O3—C33—H33A	110.3
C12—C13—H13	119.6	C34—C33—H33A	110.3
C14—C13—H13	119.6	O3—C33—H33B	110.3
C15—C14—C13	120.21 (14)	C34—C33—H33B	110.3
C15—C14—H14	119.9	H33A—C33—H33B	108.5
C13—C14—H14	119.9	C33—C34—H34A	109.5
C14—C15—C16	120.85 (12)	C33—C34—H34B	109.5
C14—C15—H15	119.6	H34A—C34—H34B	109.5
C16—C15—H15	119.6	C33—C34—H34C	109.5
C15—C16—C17	121.61 (12)	H34A—C34—H34C	109.5
C15—C16—C21	119.50 (13)	H34B—C34—H34C	109.5
C17—C16—C21	118.84 (13)	O4—C35—C36	107.01 (11)
C18—C17—C16	121.22 (12)	O4—C35—H35A	110.3
C18—C17—H17	119.4	C36—C35—H35A	110.3
C16—C17—H17	119.4	O4—C35—H35B	110.3
C17—C18—C19	119.91 (13)	C36—C35—H35B	110.3
C17—C18—H18	120.0	H35A—C35—H35B	108.6
C19—C18—H18	120.0	C35—C36—H36A	109.5
C20—C19—C18	120.76 (13)	C35—C36—H36B	109.5
C20—C19—H19	119.6	H36A—C36—H36B	109.5
C18—C19—H19	119.6	C35—C36—H36C	109.5
C19—C20—C21	121.07 (12)	H36A—C36—H36C	109.5
C19—C20—H20	119.5	H36B—C36—H36C	109.5
			
C33—O3—C2—C1	173.31 (10)	C14—C15—C16—C21	−2.7 (2)
C33—O3—C2—C3	−4.73 (17)	C15—C16—C17—C18	−175.60 (13)
C10—C1—C2—O3	−175.91 (10)	C21—C16—C17—C18	2.06 (19)
C11—C1—C2—O3	−0.66 (15)	C16—C17—C18—C19	−2.2 (2)
C10—C1—C2—C3	2.16 (18)	C17—C18—C19—C20	0.5 (2)
C11—C1—C2—C3	177.41 (11)	C18—C19—C20—C21	1.36 (18)
O3—C2—C3—C4	177.47 (11)	C19—C20—C21—C16	−1.48 (17)
C1—C2—C3—C4	−0.46 (19)	C19—C20—C21—C12	173.70 (11)
C2—C3—C4—C5	−0.7 (2)	C15—C16—C21—C20	177.51 (11)
C3—C4—C5—C6	−179.75 (12)	C17—C16—C21—C20	−0.20 (17)
C3—C4—C5—C10	0.06 (19)	C15—C16—C21—C12	2.06 (17)
C4—C5—C6—C7	−179.93 (12)	C17—C16—C21—C12	−175.65 (10)
C10—C5—C6—C7	0.3 (2)	C13—C12—C21—C20	−174.76 (12)
C5—C6—C7—C8	−0.7 (2)	C11—C12—C21—C20	5.09 (17)
C35—O4—C8—C9	−177.76 (10)	C13—C12—C21—C16	0.42 (16)
C35—O4—C8—C7	3.98 (18)	C11—C12—C21—C16	−179.73 (10)
C6—C7—C8—O4	177.47 (12)	C8—C9—C22—O2	−111.50 (13)
C6—C7—C8—C9	−0.7 (2)	C10—C9—C22—O2	65.60 (17)
O4—C8—C9—C10	−175.82 (10)	C8—C9—C22—C23	66.74 (14)
C7—C8—C9—C10	2.45 (19)	C10—C9—C22—C23	−116.16 (12)
O4—C8—C9—C22	1.45 (16)	O2—C22—C23—C24	−132.46 (12)
C7—C8—C9—C22	179.73 (11)	C9—C22—C23—C24	49.32 (15)
C2—C1—C10—C9	177.29 (11)	O2—C22—C23—C32	46.38 (17)
C11—C1—C10—C9	2.59 (18)	C9—C22—C23—C32	−131.84 (11)
C2—C1—C10—C5	−2.66 (17)	C32—C23—C24—C25	−3.29 (18)
C11—C1—C10—C5	−177.36 (10)	C22—C23—C24—C25	175.57 (12)
C8—C9—C10—C1	177.30 (11)	C23—C24—C25—C26	1.5 (2)
C22—C9—C10—C1	0.36 (19)	C24—C25—C26—C27	1.4 (2)
C8—C9—C10—C5	−2.76 (17)	C25—C26—C27—C28	175.37 (13)
C22—C9—C10—C5	−179.70 (10)	C25—C26—C27—C32	−2.4 (2)
C6—C5—C10—C1	−178.61 (11)	C26—C27—C28—C29	−175.54 (13)
C4—C5—C10—C1	1.59 (17)	C32—C27—C28—C29	2.3 (2)
C6—C5—C10—C9	1.45 (17)	C27—C28—C29—C30	−1.3 (2)
C4—C5—C10—C9	−178.35 (11)	C28—C29—C30—C31	−0.7 (2)
C2—C1—C11—O1	−114.04 (12)	C29—C30—C31—C32	1.7 (2)
C10—C1—C11—O1	60.90 (16)	C30—C31—C32—C27	−0.59 (18)
C2—C1—C11—C12	64.06 (14)	C30—C31—C32—C23	175.03 (11)
C10—C1—C11—C12	−121.00 (12)	C26—C27—C32—C31	176.52 (12)
O1—C11—C12—C13	−132.64 (13)	C28—C27—C32—C31	−1.34 (17)
C1—C11—C12—C13	49.27 (15)	C26—C27—C32—C23	0.66 (18)
O1—C11—C12—C21	47.51 (16)	C28—C27—C32—C23	−177.21 (11)
C1—C11—C12—C21	−130.58 (11)	C24—C23—C32—C31	−173.48 (12)
C21—C12—C13—C14	−2.40 (19)	C22—C23—C32—C31	7.71 (17)
C11—C12—C13—C14	177.75 (12)	C24—C23—C32—C27	2.15 (17)
C12—C13—C14—C15	1.9 (2)	C22—C23—C32—C27	−176.67 (11)
C13—C14—C15—C16	0.7 (2)	C2—O3—C33—C34	179.03 (11)
C14—C15—C16—C17	175.00 (14)	C8—O4—C35—C36	177.51 (11)

### Hydrogen-bond geometry (Å, º)

*Cg*4 and *Cg*6 are the centroids of the C16–C21 and C27–C32 rings, respectively.

**Table:**

*D*—H···*A*	*D*—H	H···*A*	*D*···*A*	*D*—H···*A*
C3—H3···*Cg*4^i^	0.95	2.77	3.5662 (15)	142
C7—H7···*Cg*6^i^	0.95	2.76	3.5662 (16)	143
C30—H30···O2^ii^	0.95	2.53	3.3289 (19)	142
C34—H34*A*···O1^iii^	0.98	2.47	3.423 (2)	163
C35—H35*B*···O2^iv^	0.99	2.59	3.5476 (17)	163

Symmetry codes: (i) *x*+1, *y*, *z*; (ii) −*x*+1, −*y*+2, −*z*+2; (iii) −*x*, −*y*+1, −*z*+1; (iv) −*x*, −*y*+2, −*z*+2.
